# Trust by design in AI-augmented procurement systems: the roles of explainability, governance, and human oversight

**DOI:** 10.3389/frai.2026.1752349

**Published:** 2026-07-31

**Authors:** Raja Mejri, Sameh Skhiri

**Affiliations:** 1 University of Jeddah, Jeddah, Saudi Arabia; 2 LISEFE-Lab, Tunis El Manar University, Tunis, Tunisia; 3 Department of Marketing, Faculty of Economic and Management Sciences of Nabeul, University of Carthage, Nabeul, Tunisia

**Keywords:** AI-augmented procurement negotiation systems, ethical governance, explainable artificial intelligence, human-in-the-loop, trust in AI, relational design, structural equation modeling, supply chain management

## Abstract

With the growing integration of artificial intelligence (AI) into buyer–supplier negotiations, procurement teams must translate efficiency gains into defensible and appropriately calibrated reliance on AI-mediated decision support. This study develops and empirically evaluates a socio-technical trust-by-design model for AI-augmented procurement negotiation systems. It jointly considers perceived transparency/explainability (XAI), ethical governance visibility, and human-in-the-loop (HIL) relational design as conceptually distinct levers of AI system trust—that is, professionals’ willingness to rely on an AI negotiation system and their belief that it behaves competently and responsibly. AI system trust is treated as a focal proximal outcome, not as a proxy for, or a sufficient explanation of, dyadic buyer–supplier trust. Using an exploratory sequential mixed-methods approach—six multi-sector case studies across the EU and ASEAN regions (36 interviews) followed by a purposively recruited cross-sectional survey of 238 AI-exposed professionals analyzed via structural equation modeling (SEM)—we find that AI use is associated with higher perceived negotiation efficiency, but also with lower AI system trust when automation displaces relational cues and explanations are weak. XAI exhibits the strongest positive association with AI system trust, while HIL design and governance show additional positive associations. In an augmented specification, XAI and HIL statistically account for the AI use–trust association. A small inverse-U pattern further suggests that moderate, well-governed AI use is associated with higher trust than very low or very high automation intensity. Multi-group analyses indicate stronger XAI–trust associations in the EU and stronger HIL associations in ASEAN. These findings contribute to trust-in-AI and procurement research by (i) clarifying the boundary between trust in an AI system and trust in a buyer or supplier, (ii) specifying procurement-specific conditions—confidentiality, auditability, and negotiation tacticity—under which AI use can erode confidence in AI support, and (iii) offering a cautiously framed, evidence-consistent roadmap for trustworthy AI negotiation systems. The reported associations should be interpreted within a non-probability sample and do not establish population-representative effects or buyer–supplier relationship outcomes.

## 1 Introduction

The rapid advancement of artificial intelligence (AI) and digital technologies is transforming how organizations coordinate, communicate, and negotiate across firm boundaries. From sales automation to strategic sourcing, digital transformation is reshaping interfirm processes and the way information, value, and accountability are created and exchanged (Agnihotri, 2021; Coussement et al., 2024). Digitally enabled supply-chain integration and e-business applications have also been linked to operational performance (Rai et al., 2006; Wiengarten et al., 2011). In supply chain and operations management, recent work emphasizes that generative and predictive AI are redefining decision-making and governance structures, altering the logic of collaboration and control (Jackson et al., 2024). In information systems research, explainable AI (XAI) has become a central lever for decision legitimacy and stakeholder acceptance (Coussement et al., 2024).

Scope of the focal technology. In this paper, AI-augmented procurement negotiation systems refer to digital tools or platforms that use machine learning and/or generative models to support negotiation work by (i) producing recommendations (e.g., pricing ranges, concession paths, BATNA-related analytics, supplier risk scores), (ii) drafting or reviewing negotiation artifacts (e.g., emails, term sheets, contract clauses), and/or (iii) automating limited steps of interaction (e.g., suggestion of counter-offers), while contractual commitments and final decisions remain under human authority. This broad definition reflects the heterogeneous reality of procurement AI in practice and frames our empirical measures, which capture perceived system features rather than a single vendor-specific implementation.

Trust construct boundary and potential cross-influence. Procurement negotiations traditionally rely on interpersonal trust, relational norms, and strategic communication. As these interactions become AI-mediated, trust can refer to (a) interfirm relational trust in the supplier or buyer counterpart or (b) AI system trust in the artifact that supports negotiation decisions. The present study focuses on AI system trust—professionals’ willingness to rely on an AI negotiation system and their belief that it behaves competently, fairly, and responsibly—because this construct aligns with the measurement items concerning recommendations, perceived integrity, and protection of sensitive information. This analytical distinction does not imply causal independence. Trust in the AI may shape whether recommendations are enacted and how offers, concessions, or messages are communicated; conversely, pre-existing trust in the counterpart may influence whether AI-supported advice is accepted or contested. Because the survey did not measure dyadic trust or matched buyer–supplier perceptions, these reciprocal pathways cannot be estimated here. AI system trust is therefore treated as the focal, not exhaustive or sufficient, dependent variable, and we do not infer cooperation or relationship quality from it alone (Lee and See, 2004; Zaheer et al., 1998).

While AI offers substantial benefits—shorter decision cycles, enhanced market intelligence, and the potential to mitigate some forms of human bias—it also introduces managerial, ethical, and strategic challenges. Concerns increasingly center on opacity, contested accountability, and erosion of socio-cognitive cues that typically support appropriate reliance (Wanner et al., 2022; Riedl, 2022). These challenges are particularly salient in procurement negotiations, where decisions are high-stakes, information is sensitive, and parties may have partially misaligned incentives. Recent international-business research likewise emphasizes the opportunities and organizational challenges associated with AI adoption (Menzies et al., 2024; Ratten et al., 2024).

Why procurement and negotiation are theoretically distinctive. Established research on trust in automation and trust in AI highlights the importance of transparency, governance, and human oversight for appropriate reliance. However, procurement negotiations create boundary conditions that can change the sign and strength of these relationships: confidentiality and data-leakage risks, auditability requirements, contractual incompleteness, power asymmetries, and negotiation tacticity (i.e., the strategic sensitivity of offers and concessions). In such contexts, increasing automation can accelerate processes yet reduce confidence in AI outputs if the system’s reasoning cannot be defended, contested, or overridden in ways that are acceptable to internal stakeholders and external counterparts.

Building on negotiation-support research (Kersten and Noronha, 1999; Kersten and Lai, 2007), AI ethics and governance debates (OECD AI Principles; EU AI Act), and Digital Market Design (DMD) perspectives on rule visibility and contestability, we develop a socio-technical trust-by-design framework that jointly considers three conceptually distinct levers: perceived transparency/explainability (XAI), ethical governance visibility, and human-in-the-loop relational design. The empirical model estimates their unique main-effect associations with AI system trust; it does not assume or test that the levers are interchangeable, strictly additive in a causal sense, or mutually compensatory.

We test the framework using an exploratory sequential mixed-methods design (QUAL → QUAN): six cross-regional case studies (36 interviews) inform construct refinement and survey-item development, followed by a cross-sectional survey of 238 professionals analyzed via SEM.

Because our quantitative evidence is cross-sectional and single source, we interpret estimated relationships as associations rather than causal effects. Accordingly, our design implications are presented as propositions consistent with the evidence and with existing theory, not as time-ordered prescriptions.

Regulatory developments further motivate the study. The EU Artificial Intelligence Act—entered into force on August 1, 2024—phases in obligations for prohibited practices, general-purpose AI, and high-risk systems. We therefore discuss how a sequenced set of trust-by-design actions (e.g., audit logs and human review gates, explainability and data provenance, and risk-management documentation) can be aligned with these regulatory timelines. Importantly, this roadmap is derived from policy requirements and our empirical patterns; it should be read as an evidence-consistent implementation heuristic, not a causal claim.

This study makes three contributions. First, it clarifies AI system trust as a focal proximal outcome, distinguishes it from dyadic buyer–supplier trust and relationship quality, and explicitly identifies possible cross-influence between these trust targets as an unmeasured boundary condition. Second, it specifies procurement-specific mechanisms and conditions—confidentiality, auditability, and negotiation tacticity—that help explain why AI use can increase efficiency yet erode confidence in AI support, and it documents a non-linear (inverse-U) pattern consistent with this tension. Third, it provides mixed-methods evidence across two institutional contexts (EU and ASEAN), offering context-sensitive propositions for trust-by-design while limiting generalization to the sampled AI-exposed professionals.

The remainder of the paper proceeds as follows. Section 2 reviews the theoretical foundations and develops hypotheses linking AI use to perceived efficiency and AI system trust through transparency, governance, and human oversight mechanisms. Section 3 describes the exploratory sequential mixed-methods design. Section 4 presents the results and integrates qualitative and quantitative insights. Section 5 concludes with contributions, implications, limitations, and directions for future research on trust in AI-mediated procurement negotiations.

## 2 Literature review and hypotheses development

Artificial intelligence (AI) is increasingly embedded in procurement and sourcing work, including the preparation, conduct, and documentation of B2B negotiations. Negotiation-support and e-negotiation systems have long shown how digital tools reshape the pace, information visibility, and structure of bargaining (Kersten and Noronha, 1999; Kersten and Lai, 2007). Contemporary AI—ranging from predictive analytics to generative negotiation assistants—extends these effects by altering informational and procedural asymmetries, raising new questions about appropriate reliance and trust.

### 2.1 Trust in automation and AI systems

Trust in technology has been extensively studied in the trust-in-automation and trust-in-AI literatures. A core insight is that system design should foster appropriate reliance—calibrated trust that reflects system competence, limitations, and the user’s ability to monitor and intervene (Lee and See, 2004). Reviews further emphasize that trust judgments are shaped by perceived competence, integrity, transparency, and controllability, and that trust can be fragile when systems are opaque or difficult to contest (Hoff and Bashir, 2015; Glikson and Woolley, 2020; Choung et al., 2023; Rodriguez Rodriguez et al., 2023). Trust also shapes users’ acceptance of online and AI-enabled systems (Gefen et al., 2003; Lukyanenko et al., 2022).

In AI-mediated procurement negotiations, these insights translate into a focus on AI system trust: a professional’s willingness to rely on an AI negotiation system and to believe that the system will behave competently and responsibly during negotiation support. The construct is analytically distinct from interfirm relational trust because the object of reliance differs—the AI artifact versus the buyer or supplier counterpart. Analytical distinction, however, does not imply causal independence: AI system trust can influence whether advice is enacted and communicated, while pre-existing counterpart trust can shape whether AI-supported recommendations are accepted, challenged, or interpreted as legitimate. The present model focuses on AI system trust because it matches the research question and measurement instrument; it does not treat AI system trust as sufficient to explain cooperation or relationship quality.

Measurement implication. Because dyadic buyer–supplier trust was not included in the survey, the study cannot estimate reciprocal or spillover paths between the two trust targets. The CFA and discriminant-validity tests reported below establish separation among AI system trust and the measured constructs (AIU, XAI, EG, RD, and PE), but they cannot demonstrate independence from an unmeasured relational-trust construct. This boundary motivates the dyadic designs proposed in Section 5.

### 2.2 Why procurement negotiations are a distinctive trust context

Procurement negotiations differ from many decision-support settings where trust in AI has been studied. First, negotiation decisions are high-stakes and often contestable (internally and externally): procurement teams must justify offers, concessions, and contract clauses to legal, finance, compliance, and sometimes regulators. Second, negotiations involve sensitive information (prices, margins, volumes, strategy) and confidentiality risks that heighten concerns about data leakage and misuse. Third, procurement relationships are embedded in power asymmetries and contractual incompleteness, making fairness perceptions and accountability central to legitimacy.

These conditions create a distinctive tension: increasing AI use may be associated with higher efficiency while also being associated with lower AI system trust if the system’s reasoning is opaque, if governance is invisible, or if human agency is weakened. This procurement-specific “efficiency–trust tension” provides a theoretical reason why the sign of the AI-use → trust association can differ from more cooperative or low-stakes contexts.

### 2.3 Trust-by-design levers and construct boundaries

To explain how AI system trust can be fostered in negotiation contexts, we jointly consider three levers located at distinct socio-technical layers. Interfirm trust is central to buyer-seller relationships and relationship marketing (Doney and Cannon, 1997; Palmatier et al., 2006). The hypotheses below concern their unique main effects. Possible interactions—substitution, compensation, or synergy—are theoretically plausible but are not estimated in the present model.

1. Transparency and explainability (XAI). XAI refers to users’ perceived ability to understand why the system produced a recommendation and to trace the data and decision logic underpinning it. In procurement negotiations, explainability supports perceived competence and procedural fairness and helps users defend AI outputs in audit-oriented settings (Wanner et al., 2022; Coussement et al., 2024).
2. Ethical governance visibility. Ethical governance refers to visible oversight, accountability, auditability, and redress mechanisms surrounding the AI system (e.g., ethics/compliance review, reporting channels, documentation, and escalation procedures). Governance visibility signals integrity and legitimacy, and it reduces ambiguity about responsibility when outcomes are disputed (Jorzik et al., 2024; Organisation for Economic Co-operation and Development, 2019; European Union, 2024).
3. Human-in-the-loop relational design (HIL/RD). Relational design captures interface- and process-level safeguards that preserve human agency and socio-affective cues during AI-mediated interactions (e.g., explicit review gates, override authority, role clarity, and human contact channels). In negotiation settings—where tactics, tone, and relationship cues matter—human oversight increases controllability and mitigates depersonalization risks.

Construct clarity, non-redundancy, and interaction boundary. XAI concerns the intelligibility of model outputs; governance concerns the organizational and institutional accountability structure surrounding the system; and relational design concerns the interaction architecture that enables monitoring, intervention, and socio-affective continuity. These levers address different bases of trust. XAI can make an output intelligible but cannot by itself assign responsibility or provide redress; governance can allocate accountability but cannot make an individual recommendation understandable; and HIL design permits intervention but does not itself establish explanation or institutional legitimacy. Accordingly, no lever is assumed to fully compensate for the absence of another. At the same time, the marginal effect of one lever may vary with the level of another. The present study estimates unique main effects only and therefore does not test substitution, compensation, or synergy among XAI, governance, and HIL/RD.

### 2.4 Digital market design and rule transparency in procurement platforms

Digital Market Design (DMD) research conceptualizes digital exchange environments as engineered rule systems that structure information flows, interaction protocols, and enforcement mechanisms (Roth, 2015). From this perspective, AI-augmented procurement negotiation systems can be understood as rule-governed marketplaces in which transparency is not only model-level (why a recommendation) but also rule-level (under which rules it is produced, prioritized, audited, and contested).

We therefore conceptualize market-rule transparency as the clarity, accessibility, and verifiability of the “rulebook” governing AI-mediated negotiation platforms across five domains: (i) access rules (rights and participation), (ii) information-disclosure rules (explanations and data provenance), (iii) interaction rules (HIL checkpoints and escalation), (iv) allocation/pricing rules (how proposals are generated or prioritized), and (v) enforcement/appeal rules (accountability and redress). In our empirical model, this rule transparency is reflected primarily through perceived XAI, ethical governance visibility, and relational design.

### 2.5 Hypotheses

Building on the logic above, we propose four main-effect hypotheses linking AI use to perceived efficiency and AI system trust through technical (XAI), institutional (governance), and relational (HIL) mechanisms.

*H1*: In B2B procurement negotiations, higher AI use is associated with higher perceived operational efficiency, but associated with lower AI system trust when transparency and human safeguards are insufficient.

Rationale. AI can accelerate information processing and reduce cycle time, yet it can also depersonalize interactions and reduce defensibility of recommendations when explanations and human oversight are weak (Kersten and Lai, 2007; Corsaro and D’Amico, 2022).

*H2*: In AI-augmented negotiations, higher perceived transparency and explainability (XAI) is associated with higher AI system trust.

Rationale. Explanations and traceability increase perceived competence and fairness and support calibrated reliance in intelligent systems (Lee and See, 2004; Wanner et al., 2022; Coussement et al., 2024).

*H3*: Greater ethical governance visibility—clear accountability, auditability, and redress—is associated with higher AI system trust.

Rationale. Visible governance structures and risk-based oversight increase legitimacy signals and reduce ambiguity about responsibility in AI-mediated decisions (Jorzik et al., 2024; Organisation for Economic Co-operation and Development, 2019; European Union, 2024).

*H4*: Stronger human-in-the-loop relational design (HIL/RD) is associated with higher AI system trust.

Rationale. Human oversight and socio-affective design increase controllability and preserve negotiation-relevant relational cues, mitigating trust erosion as automation increases (Morgan and Hunt, 1994; Hoff and Bashir, 2015).

**Figure 1:**
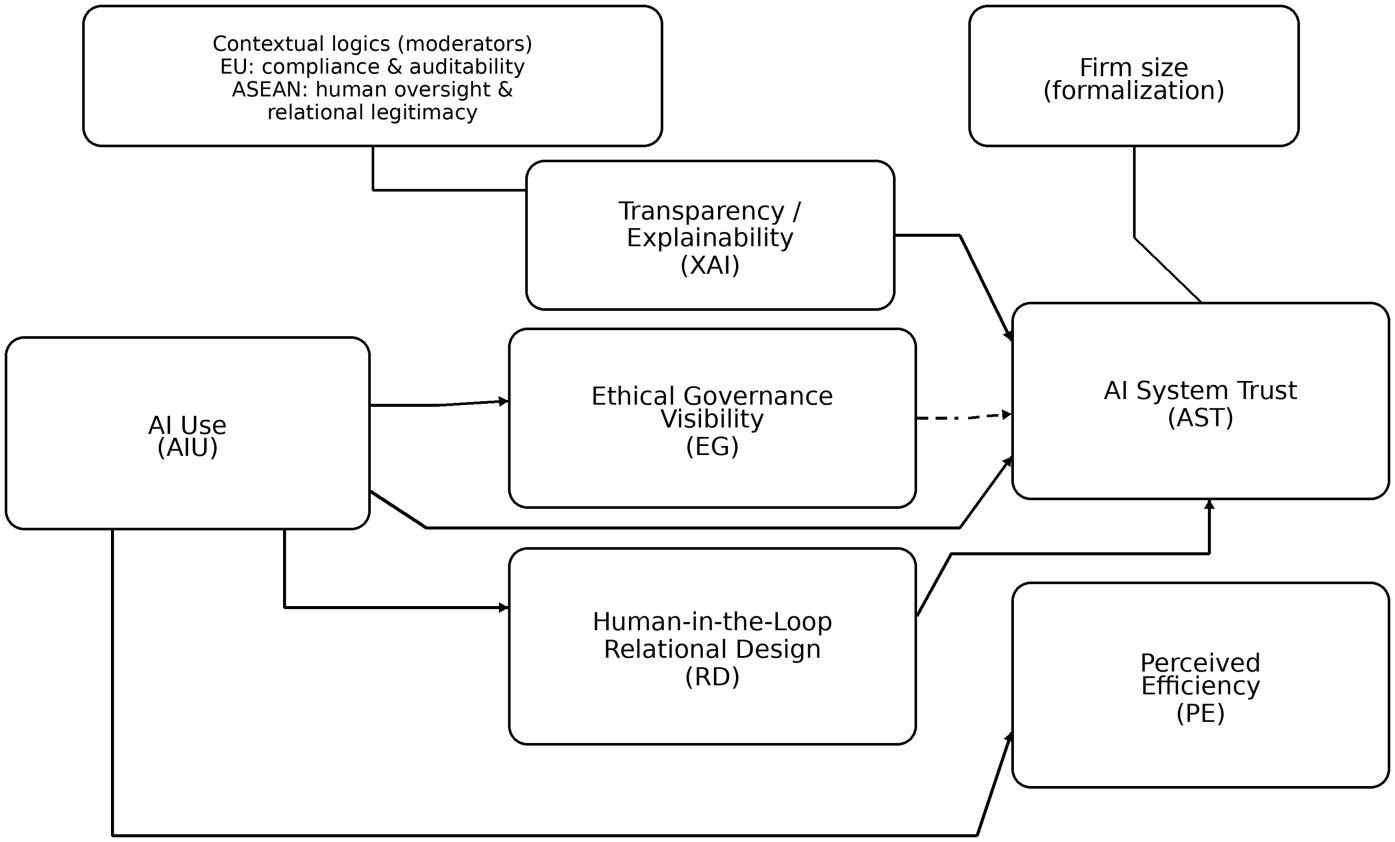
Digital market design framework for AI-augmented procurement negotiations: rule transparency, XAI, human oversight, and AI system trust.

Figure 1 summarizes our socio-technical market-design framework and the hypothesized paths (H1–H4).

The framework treats AI-augmented procurement negotiations as a digital-market design problem. Market-rule transparency operationalizes governance at the rule level and is reflected through explainability (XAI), human-in-the-loop (HIL) interaction rules, and visible ethical governance (accountability and redress). AI use is expected to be associated with higher perceived efficiency but may be associated with lower AI system trust when automation intensity increases without sufficient explanations and human safeguards. Contextual conditions (EU logic: compliance and auditability; ASEAN logic: human oversight and relational legitimacy) shape the relative salience of XAI versus HIL. The solid paths represent main-effect expectations; interactions among XAI, governance, and HIL/RD are not estimated.

## 3 Method

We employ an exploratory sequential mixed-methods design (QUAL → QUAN) grounded in a pragmatic paradigm to examine how organizations build and sustain trust in AI-augmented B2B procurement negotiations (Creswell and Plano Clark, 2018). The qualitative phase identifies mechanisms and refines constructs, while the subsequent quantitative phase tests whether explainability (XAI), ethical governance, and human-in-the-loop relational design are associated with AI system trust.

### 3.1 Research design and context

**Figure 2:**
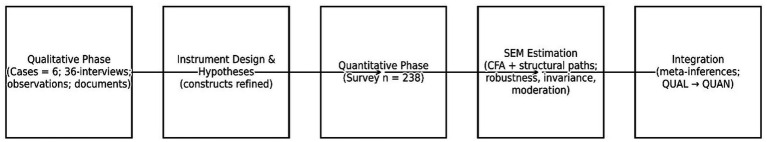
Exploratory sequential mixed-methods design (QUAL → QUAN).

The study unfolds in two integrated phases: multiple case studies inform construct definitions and hypotheses, and a cross-regional survey empirically tests the structural model. The empirical context covers procurement and sourcing functions in medium- to large-sized organizations operating in contract-intensive sectors—manufacturing, healthcare, finance, and information technology—where interfirm trust and relationship governance strongly influence performance (Zaheer et al., 1998). Two regions capture institutional and cultural variation: the European Union (EU), characterized by compliance, traceability, and stringent data-protection regimes (e.g., GDPR); and Southeast Asia (ASEAN), marked by rapid adoption and efficiency under fragmented regulatory oversight (Mügge, 2024). This design permits contextual comparison and supports analytical transferability rather than statistical representativeness (Yin, 2018). Figure 2 summarizes the sequential process linking qualitative exploration, quantitative testing, and subsequent integration.

### 3.2 Qualitative phase

Six organizations were examined using maximum-variation sampling (Yin, 2018): three in Europe (utilities, retail distribution, and public health) and three in Southeast Asia (manufacturing, finance, and public administration). Data triangulation combined 36 semi-structured interviews (45–75 min) with procurement executives, legal counsel, and digital-transformation managers; observations of negotiation simulations; and document analysis of ethics protocols and platform logs (Miles et al., 2014). NVivo-assisted thematic analysis, conducted independently by two coders (*κ* = 0.82), combined deductive categories—transparency/XAI, trust, governance, and relational design—with inductive coding to capture emergent concerns (e.g., relational displacement, affective signaling, and power asymmetries). Coding proceeded iteratively (open coding → axial grouping → cross-case comparison), and analytic memos were used to link qualitative patterns to construct refinements and survey item wording. Four cross-case patterns emerged, informing construct refinement, survey-item development, and EU–ASEAN multi-group analysis: (1) the centrality of explainability/XAI for defensible reliance; (2) the displacement and depersonalization of interpersonal exchanges; (3) ethical tensions and perceived power asymmetries when AI access is unequal; and (4) governance-visibility gaps that weaken accountability signals. To enhance mixed-methods transparency, Appendix Table A4 provides an audit trail linking representative qualitative evidence to construct decisions, item origins (validated vs. case-derived), and hypothesis development.

### 3.3 Quantitative phase

A structured survey tested the hypothesized relationships among AI Use (AIU), Transparency/Explainability (XAI), Ethical Governance (EG), Relational Design (RD), Perceived Efficiency (PE), and AI System Trust (AST). Measures combined validated scales with items derived from the qualitative phase, using five-point Likert responses (1 = strongly disagree; 5 = strongly agree). The final sample comprised 238 respondents: procurement managers (55%), contract/legal specialists (23%), and digital-transformation managers (22%). Of these, 54% were based in the EU and 46% in ASEAN, representing manufacturing (40%), healthcare (27%), finance (21%), and IT (12%). Data were collected between March and July 2024. Inclusion criteria required respondents to work in procurement/contracting, legal/compliance, or digital-transformation roles with exposure to AI-supported procurement processes.

Sampling and inferential scope. Recruitment used purposive convenience access through (i) professional procurement and supply-chain networks, (ii) executive-education and academic partnership channels, and (iii) targeted outreach to practitioners involved in negotiation and contracting. Because the survey link was distributed through multiple overlapping channels and participation was anonymous, an exact response rate could not be computed. The resulting sample is non-probability and cannot be considered statistically representative. Professionals with greater interest in, experience with, or favorable attitudes toward AI may have been more likely to participate, whereas nonparticipants and less AI-engaged practitioners are systematically unobserved. Cross-regional, cross-sector, and cross-role heterogeneity broadens empirical coverage but does not eliminate self-selection or non-response bias. No sampling frame was available for weighting or respondent–nonrespondent comparison. Accordingly, conclusions concern associative patterns among the sampled AI-exposed professionals rather than population prevalence or population-average effect sizes. Firm size, digital maturity, negotiation frequency, and region are included as controls, but these covariates do not correct selection into the sample.

### 3.4 Measurement and estimation

All focal constructs were modeled as latent reflective variables (AIU, XAI, EG, RD, PE, AST). The survey instrument was reviewed by three professors in information systems and procurement for content validity, and a pilot test with 15 professionals confirmed clarity before full deployment. Conceptually, Relational Design (RD) comprises two complementary facets: human-in-the-loop (HIL) safeguards (oversight and override) and socio-affective cues (interpersonal signaling during interactions). Our main model treats RD as a single reflective factor (RD1–RD4); a two-factor specification (RD-HIL / RD-CUES), potentially with an Interface Quality (UIQ) control, is outlined as a robustness extension and reserved for future analyses to avoid over-extending the present empirical scope. Models were estimated using structural equation modeling (SEM) in AMOS 28. A confirmatory factor analysis (CFA) verified measurement quality (Hair et al., 2021): Cronbach’s *α* and composite reliability > 0.80; average variance extracted > 0.50; HTMT ###### 0.85; and Fornell–Larcker criteria satisfied. To address potential common-method variance, we applied procedural remedies (anonymity, varied scale anchors) and conducted both Harman’s single-factor test and a latent-method-factor comparison; neither indicated a dominant single factor or significant improvement in model fit (|ΔCFI| ≤ 0.01). Robustness checks included 5,000-sample bootstrapping, multi-group measurement invariance (configural, metric, scalar; EU vs. ASEAN), and moderation tests (Digital Maturity × XAI → AST; Firm Size × EG → AST). All variance inflation factors (VIF) were below 3.0, suggesting no multicollinearity issues. Model-fit thresholds followed Hu and Bentler (1999): CFI > 0.90; TLI > 0.90; RMSEA ###### 0.06; SRMR ###### 0.08. Detailed CFA results appear in Section 4, Table 1. The XAI → Trust association may be biased by reverse causality (trust-oriented organizations investing more in explainability). We mitigate this risk by: (i) controlling for firm size, digital maturity, negotiation frequency, and region; (ii) replicating the sign pattern and relative magnitudes using PLS-SEM (Appendix B); and (iii) testing a latent method factor (with |ΔCFI| ≤ 0.01) to probe common-method variance. In line with a cautious interpretation, we therefore refer to associations rather than strict causal effects. Looking ahead, future research could strengthen identification by developing an instrumental-variables approach based on sectoral regulatory pressure (e.g., NACE exposure to high-risk AI use cases). Conceptually, such an instrument could combine industry-level exposure to “high-risk” applications—derived from NACE two-digit codes and the EU AI Act risk taxonomy—with proxies of compliance intensity such as ISO certifications or audit requirements. This design would capture exogenous variation in explainability pressures without directly influencing AI system trust, thus meeting the relevance and exclusion conditions in principle.

**Table 1:** Construct reliability and validity (CFA).

Construct	α	CR	AVE	Transparency/XAI	Ethical governance	Relational design	AI system trust	Perceived efficiency
Transparency/Explainability (XAI)	0.89	0.91	0.63	—				
Ethical governance	0.83	0.87	0.58	0.46	—			
Relational design	0.85	0.88	0.61	0.49	0.42	—		
AI system trust	0.91	0.93	0.66	0.64	0.48	0.52	—	
Perceived efficiency	0.87	0.89	0.62	0.51	0.39	0.46	0.44	—

α = Cronbach’s alpha; CR = composite reliability; AVE = average variance extracted. Off-diagonal elements indicate inter-construct correlations (lower triangle).

Model-scope clarification. The reported SEM is an additive main-effects specification. We did not estimate XAI × EG, XAI × RD, EG × RD, or three-way interactions; consequently, the coefficients cannot establish substitution, compensation, or synergy among the three levers. Dyadic buyer–supplier trust was also not measured, so the model does not test cross-influence between AI system trust and relational trust. The CFA evidence establishes discriminant validity only among the constructs included in the measurement model.

Model specification Perceived Efficiency (PE) is modeled as a function of AI Use (AIU), and AI System Trust (AST) is modeled as a function of AIU, Transparency / Explainability (XAI), Ethical Governance (EG), and Relational Design (RD). Control variables—firm size, digital maturity, negotiation frequency, and region—enter the model as exogenous covariates.

**Figure 3:**
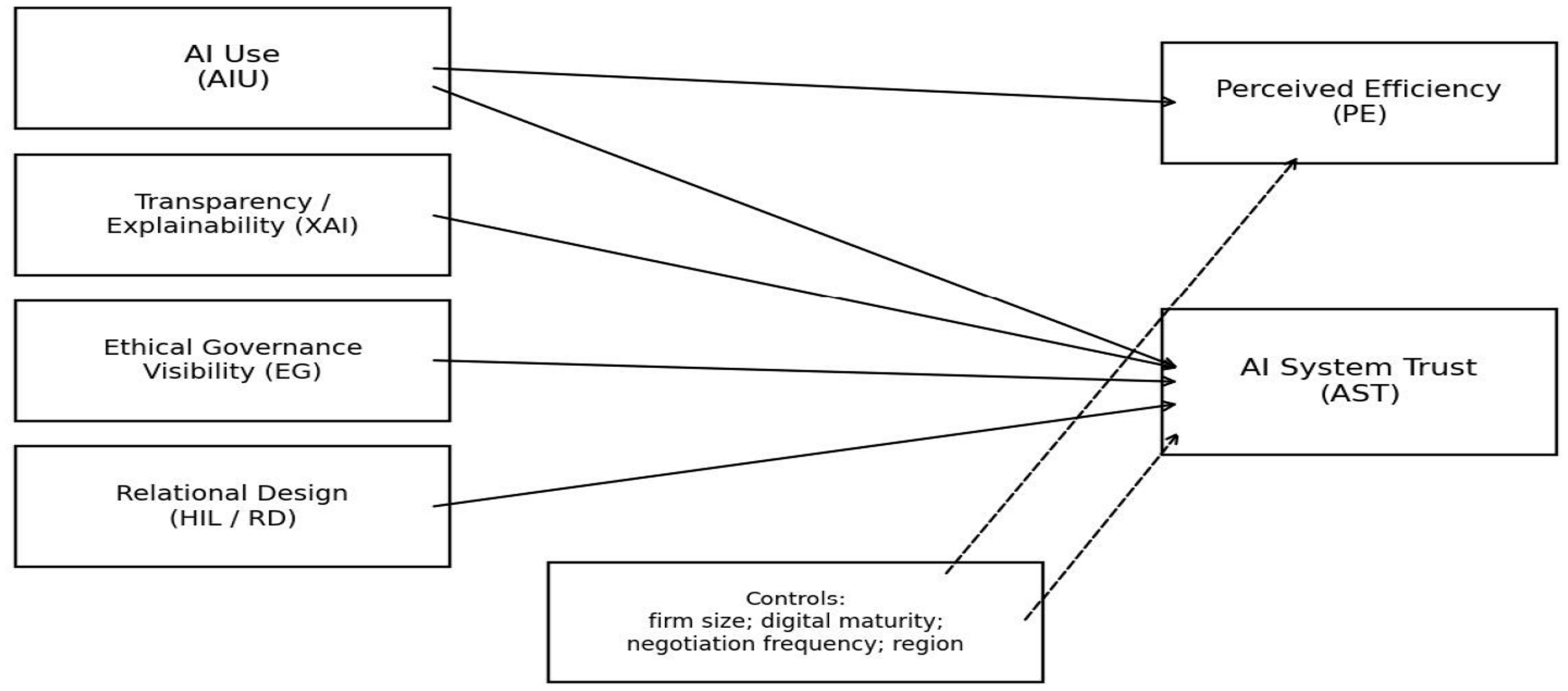
Conceptual and structural model of trust in AI-augmented B2B negotiations.

Figure 3 depicts the estimated additive main-effect paths linking AI use, transparency/explainability (XAI), ethical governance, and relational design to perceived efficiency and AI system trust. Control paths are shown as dashed arrows; pairwise and higher-order interactions among XAI, governance, and HIL/RD were not estimated.

### 3.5 Ethics and integration

All participants provided informed consent prior to data collection. The study adhered to internationally recognized ethical standards, ensuring full anonymity, confidentiality, and voluntary participation. Data were securely stored and used exclusively for academic research purposes, in line with privacy and data protection principles consistent with the EU General Data Protection Regulation (GDPR) and Institutional Review Board (IRB) guidelines. Following an exploratory-sequential logic, qualitative insights informed the conceptual definitions and hypothesis development, while the quantitative phase empirically tested these relationships. Meta-inferences then integrated the qualitative mechanisms with the statistical results, providing a coherent explanation of trust formation in AI-mediated negotiations. These procedures establish the empirical foundation for hypothesis testing. Section 4 first evaluates the measurement model (CFA; Table 1) and subsequently estimates the structural model (Figure 3) to assess hypotheses H1–H4.

## 4 Results and discussion

This section reports the empirical findings and their implications for theory and practice. It integrates evidence from the qualitative cases and the survey to explain how explainability (XAI), ethical governance, and human-in-the-loop relational design shape trust in AI-augmented B2B procurement negotiations.

### 4.1 Qualitative results

**Figure 4:**
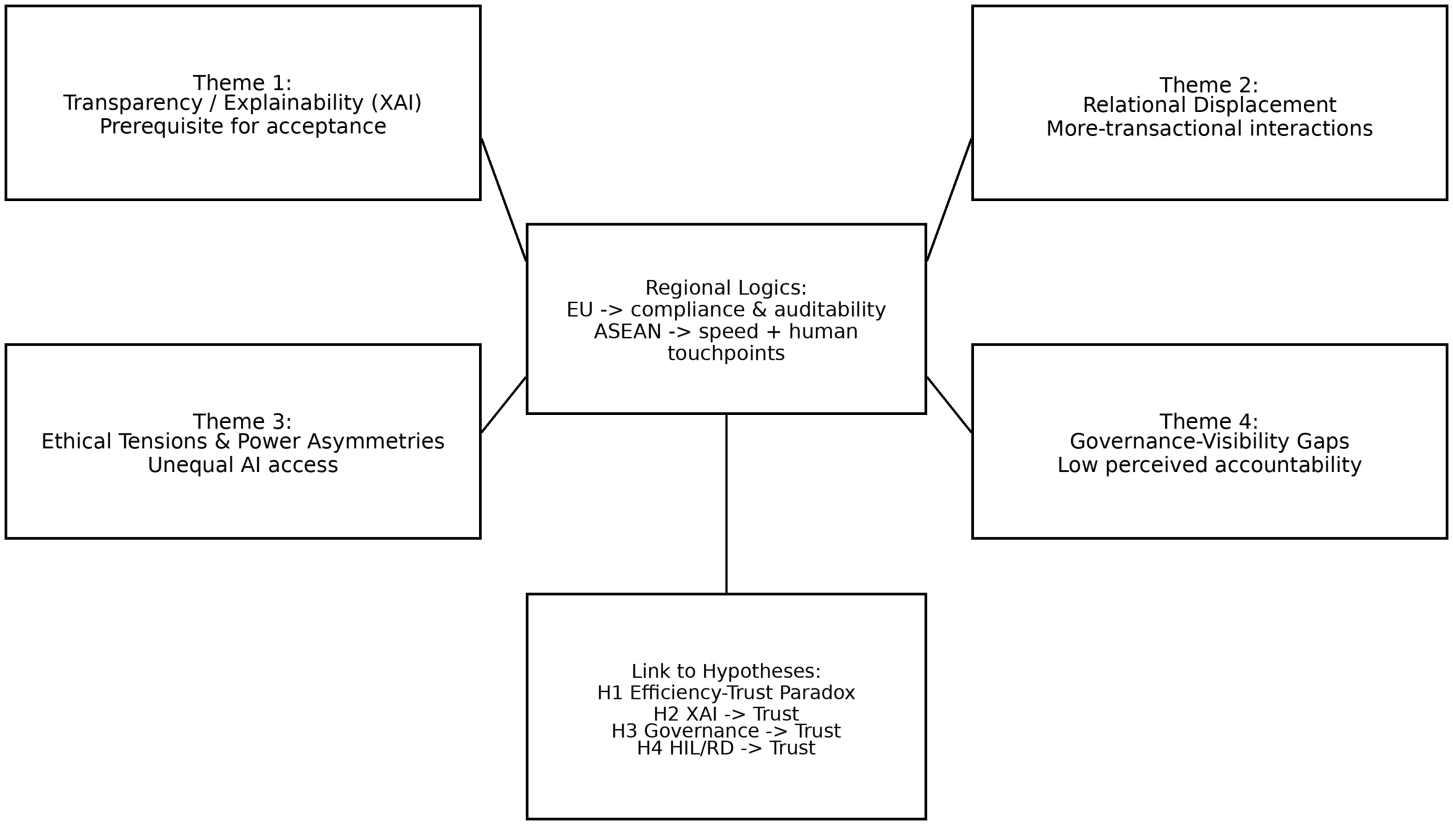
Qualitative findings and thematic framework.

Across the six organizational cases, trust did not arise automatically from efficiency gains enabled by automation. It depended on a balance between technical performance, system explainability, and the preservation of relational and ethical safeguards. Four themes recurred across cases. Transparency and explainability. In every case, systems perceived as opaque weakened trust even when outcomes were favorable. As one European procurement manager put it, “It’s not just about closing a deal, but understanding how the system got there,” underscoring explainability as a prerequisite for acceptance (Wanner et al., 2022; Riedl, 2022). Relational displacement. In five organizations, automated interactions displaced interpersonal exchanges, reducing engagement and rendering relationships more transactional, consistent with the erosion of relational capital noted by Corsaro and D’Amico (2022). Ethical tensions and power asymmetries. Several ASEAN participants described inequities when only one party possessed AI capabilities—especially in public tenders and cross-border negotiations—raising concerns about fairness and inclusiveness (Reier Forradellas and Garay Gallastegui, 2021). Governance-visibility gaps. Although most organizations had ethics or compliance committees, their actions were largely invisible to users, limiting credibility as accountability mechanisms (Mügge, 2024). A clear regional contrast emerged. In the European Union, trust hinged on compliance, auditability, and regulatory alignment—a compliance-first logic. In Southeast Asia, trust centered on efficiency and responsiveness but was sustained through human touchpoints that preserved relational legitimacy. These distinctions motivate the multi-group tests reported in §4.2. Figure 4 synthesizes the four themes, the EU/ASEAN logics, and their links to hypotheses H1–H4.

### 4.2 Quantitative results

To evaluate these insights empirically, a survey of 238 professionals was analyzed in two stages: measurement validation and structural-model estimation. The sample included respondents across regions, sectors, and roles (EU 54%; ASEAN 46%; Table 2), but it should not be interpreted as statistically representative (see Section 3.3).

**Table 2:** Sample characteristics (*n* = 238).

Variable	Categories	Percentage (%)
Region	European Union	54
	Southeast Asia	46
Sector	Manufacturing	40
	Healthcare	27
	Finance	21
	Information Technology (IT)	12
Function	Procurement	55
	Legal	23
	Digital Transformation	22
Firm size	Medium (###### 5,000 employees)	48
	Large (≥ 5,000 employees)	52

The measurement model showed satisfactory psychometrics (Table 1): Cronbach’s α and composite reliability > 0.80, AVE > 0.50, and discriminant validity supported by Fornell–Larcker and HTMT ###### 0.85.

The baseline structural model exhibited good fit (χ^2^/df = 1.94; CFI = 0.936; TLI = 0.921; RMSEA = 0.042; SRMR = 0.048). Table 3 summarises the hypothesis tests in a format fully aligned with H1–H4: H1 represents a dual prediction, reported on a single row with two coefficients (one for efficiency, one for trust), while H2–H4 correspond to explainability, ethical governance, and relational design, respectively. The hypothesized associations are supported overall, with H3 showing a smaller (“moderate”) effect size and higher contextual dependence. Multi-group analyses indicate that the effect of XAI on trust is stronger in the EU, the effect of relational design is stronger in ASEAN, and the influence of ethical governance is amplified in large firms with more formalised oversight structures (Tables 4, 5).

**Table 3:** Structural model results and group nuances (baseline SEM).

Hypothesis	Paths tested (standardized)	β → Perceived efficiency	*p*	*β* → AI system trust	*p*	Verdict	Group nuances/notes
H1	AI use → Perceived efficiency and AI use → AI system trust	0.58	######0.001	−0.41	0.006	Supported (efficiency ↑; trust ↓)	Neg. path stronger in EU (−0.47) vs. ASEAN (−0.35)
H2	Transparency/Explainability (XAI) → AI system trust	—	—	0.64	######0.001	Supported	Stronger in EU (0.71) vs. ASEAN (0.58)
H3	Ethical governance → AI system trust	—	—	0.34	0.021	Partially supported	Amplified in large firms (interaction β = 0.21, *p* ###### 0.05)
H4	Relational design (human-in-the-loop) → AI system trust	—	—	0.52	0.009	Supported	Stronger in ASEAN (0.61) vs. EU (0.47)

The model explained 34% of perceived efficiency and 61% of AI system trust.

**Table 4:** Model fit indices.

Index	Obtained value	Recommended threshold
χ^2^/df	1.94	###### 3.0
CFI	0.936	> 0.90
TLI	0.921	> 0.90
RMSEA	0.042	###### 0.06
SRMR	0.048	###### 0.08

**Table 5:** Explained variance (R^2^).

Dependent variable	R^2^
Perceived efficiency	0.34
AI system trust	0.61

To probe mechanisms and shape, we augmented the model with two mediators (XAI, Relational Design) and a quadratic term (AIU^2^). Bias-corrected bootstraps (5,000 samples) show full mediation of AI use → trust through XAI and Relational Design (direct c′ non-significant), and a negative quadratic term consistent with an inverse-U: moderate AI use favors trust, whereas high intensity erodes it (depersonalization, opacity). The augmented model improves fit (Δχ^2^ = 24.1, Δdf = 3, *p* ###### 0.001; ΔCFI = +0.015). Detailed estimates appear in Table 6.

**Table 6:** Extended model: mediation, non-linearity, and fit improvement.

Effect/path (BCa 5,000 bootstraps)	β	SE	*p*	95% CI	Interpretation
AI Use → XAI → AI system trust	0.22	0.07	0.002	[0.09; 0.37]	Significant indirect via XAI
AI Use → relational design → AI system trust	0.09	0.04	0.024	[0.02; 0.18]	Significant indirect via RD
Total indirect (XAI + RD)	0.31	0.08	######0.001	[0.18; 0.50]	Combined mediation
Direct (c′) AI use → AI system trust	0.02	0.07	0.77	[−0.12; 0.16]	Non-significant → full mediation
Quadratic AI use^2^ → AI system trust	−0.11	0.05	0.023	[−0.21; −0.02]	Inverse-U (peak ≈ 0.23 SD of AIU)
Fit (baseline → augmented)	χ^2^/df: 1.94 → 1.89 CFI: 0.936 → 0.951 TLI: 0.921 → 0.938 RMSEA: 0.042 → 0.038 SRMR: 0.048 → 0.044				Improved fit (Δχ^2^ = 24.1, Δdf = 3, *p* ###### 0.001; ΔCFI = +0.015)

### 4.3 Integrated discussion

Evidence from both phases converges on a consistent pattern: AI adoption is associated with higher negotiation efficiency, while durable AI system trust is observed when technical performance is paired with explainability, visible governance, and human-in-the-loop safeguards. In the baseline SEM, AI use is associated with higher perceived efficiency (*β* = 0.58; R^2^ = 0.34) but is negatively associated with AI system trust (β = −0.41), echoing the qualitative accounts of depersonalisation and “black-box” anxiety. Once the mechanisms are modelled explicitly, however, the story becomes more nuanced. In an augmented model that adds mediating paths from AI use to trust through explainability (XAI) and relational design (human-in-the-loop), the direct AI→trust effect becomes statistically indistinguishable from zero, while the indirect effects via XAI (β = 0.22, *p* = 0.002) and via relational design (β = 0.09, *p* = 0.024) are positive and significant; the total indirect equals 0.31 (*p* ###### 0.001). These results are statistically consistent with a full mediation pattern: the association between AI use and AI system trust operates primarily through transparency and human-in-the-loop safeguards, rather than through AI intensity per se (Appendix B, robustness tables). Shape matters as well. Adding a quadratic term reveals a small but significant inverse-U between AI use and trust (β_AI^2^ = −0.11, *p* = 0.023), with a turning point at a moderate usage level (≈ 0.23 SD of AI use). AI system trust is higher with adoption up to this point—where transparency and human oversight remain salient—then declines as automation dominates interactions. This pattern reconciles the baseline negative association with the mediated, mechanism-driven gains: when XAI and human touchpoints are weak, higher automation is associated with lower AI system trust; when both levers are strong, AI adoption is more compatible with maintaining trust in the AI negotiation system. In our measurement, Relational Design (RD) captures both human-in-the-loop safeguards and socio-affective cues, with RD3 reflecting interpersonal signaling rather than mere interface quality. This conceptual separation is further detailed in the prospective extension (RD5–RD8 and UIQ) documented in Appendix A1, which provides a basis for future replication and refinement of relational mechanisms in AI-mediated negotiations. Cross-regional comparisons are warranted because measurement invariance holds at configural, metric, and scalar levels, allowing meaningful path comparisons (Appendix B). Consistent with institutional logics, explainability carries more weight in the EU, whereas relational design is more influential in ASEAN; governance effects strengthen with firm size, where oversight is formalised and visible. All findings are robust to alternative estimators and to common-method checks (Appendices A, B).

**Figure 5:**
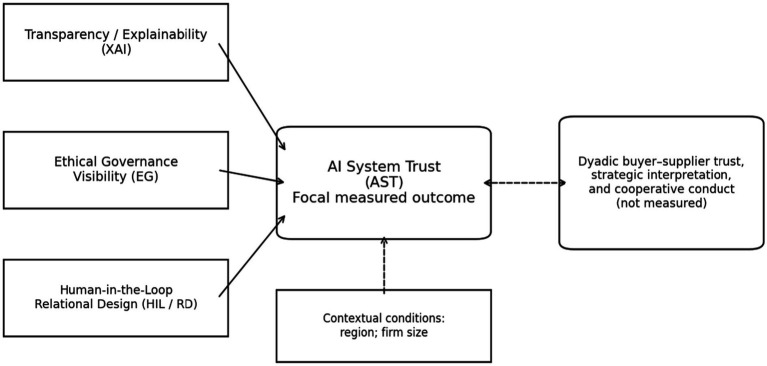
Integrated trust-boundary framework: measured AI system trust and unmeasured dyadic trust.

Trust-target boundary and plausible cross-influence. The reported coefficients explain variance in AI system trust, not the negotiator’s trust in the buyer or supplier. Low AI system trust may lead users to discount AI advice or communicate it hesitantly; excessive or poorly calibrated AI system trust may encourage overreliance and reduced scrutiny of counterpart tactics. Conversely, pre-existing dyadic trust may shape whether AI-supported recommendations are accepted or interpreted as legitimate. These pathways are plausible in light of calibrated-reliance and interorganizational-trust theory (Lee and See, 2004; Zaheer et al., 1998), but they are not identified by the present cross-sectional, single-respondent design. Figure 5 therefore represents them as an untested relational extension rather than as empirical results.

Interplay among the design levers. The positive coefficients for XAI, ethical governance, and relational design are unique main effects in a joint model. They do not show that the effects are strictly additive or that strong XAI can compensate for weak governance or HIL. Conceptually, the levers address different trust bases—intelligibility, accountability/redress, and human control/relational continuity. Substitution, compensation, or synergy requires explicit interaction tests, ideally in a powered factorial, longitudinal, or field-experimental design.

Figure 5 separates the empirically tested main-effect pathways from an unmeasured relational extension. Solid arrows represent the estimated associations of XAI, ethical governance, and HIL/RD with AI system trust. The dashed bidirectional arrow represents possible cross-influence between AI system trust and dyadic buyer–supplier trust, strategic interpretation, and cooperative conduct, none of which was directly measured. The figure also makes explicit that interactions among XAI, governance, and HIL/RD were not tested.

### 4.4 Theoretical and managerial implications

Theoretically, the results extend trust-in-automation research by distinguishing three bases of AI system trust: intelligibility (XAI), institutional accountability (governance), and controllability/relational continuity (HIL/RD). The coefficients support distinct main-effect pathways; they do not establish interchangeability or causal compensation. A highly explainable system may still lack accountability and redress, visible governance cannot make an opaque recommendation intelligible, and HIL authority cannot by itself guarantee either explanation or institutional legitimacy. The study therefore supports a bundle logic while leaving the form of any interaction open. Managerially, organizations should avoid treating any single lever as sufficient. Explainability, governance visibility, and meaningful review/override should be evaluated together, with emphasis adapted to context. Given the cross-sectional evidence, these are evidence-consistent design propositions rather than causal prescriptions. The inverse-U pattern further favors calibrated rather than maximal automation. Cross-regional patterns suggest greater salience of auditability and documentation in the EU and of human touchpoints in ASEAN, but the non-probability sample warrants cautious generalization. Importantly, these recommendations concern trust in the AI system; whether they improve buyer–supplier trust, cooperation, or strategic vigilance remains to be tested directly.

## 5 Conclusion

This study investigated how the integration of artificial intelligence into B2B procurement negotiations reconfigures how procurement professionals develop trust in AI negotiation systems and perceive implications for buyer–supplier interactions. Using an exploratory sequential mixed-methods design—six multi-sector case studies followed by a survey of 238 professionals—it shows that AI system trust is not an automatic by-product of automation but a focal proximal outcome associated with technical performance, explainability, visible governance, and human-centered relational design.

Empirically, within the sampled AI-exposed professionals, AI use is associated with higher perceived negotiation efficiency (β = 0.58) yet with lower AI system trust when automation depersonalizes interactions (β = −0.41). Explainability (XAI) is the strongest positive correlate of trust (β = 0.64), particularly in the European Union, where compliance and auditability are salient. Human-in-the-loop design is positively associated with trust (β = 0.52), especially in ASEAN contexts that emphasize interpersonal legitimacy and relational continuity. Ethical governance shows a moderate positive association (β = 0.34), increasing in strength where oversight mechanisms are visible and institutionalized. These results describe AI system trust and should not be read as direct evidence of buyer–supplier trust or cooperative behavior.

Theoretically, this research extends classical organizational trust models (Zaheer et al., 1998; McKnight et al., 2002) by incorporating technological and institutional dimensions of algorithmic negotiation. It advances a socio-technical conception of AI system trust that combines algorithmic performance, explainability, governance visibility, and relational safeguards across regulatory regimes. The proposed framework bridges organizational trust, explainable AI, and interorganizational governance by showing how digital and human mechanisms jointly shape perceived legitimacy and willingness to rely on AI support. It does not establish cooperation or relationship quality. Managerially, the results support explainability-by-design, visible governance, and meaningful human review and override, while cautioning that no single lever can be treated as sufficient and that interaction effects remain untested.

The research also faces limitations. First, the focal outcome is AI system trust rather than dyadic buyer–supplier trust. This is an analytical boundary, not evidence of causal independence. The study cannot test whether low AI system trust reduces favorable interpretations or willingness to cooperate, whether high AI system trust produces overreliance and reduced strategic vigilance, or whether pre-existing dyadic trust shapes reliance on AI advice. Future research should use matched buyer–supplier data, repeated observations, or cross-lagged and experimental designs to estimate these reciprocal pathways directly.

Second, XAI, ethical governance, and HIL/RD were estimated as additive main effects. No pairwise or three-way latent interactions were tested; therefore, the study cannot determine whether strong XAI reduces the marginal importance of governance, whether HIL is a necessary condition, or whether one lever compensates for another. Factorial vignette studies and field experiments should manipulate these levers independently and jointly to distinguish complementarity, substitution, and threshold effects.

Third, the survey used purposive convenience recruitment through professional and academic networks, and the absence of a sampling frame prevented calculation of a response rate or comparison with nonrespondents. The sample may therefore overrepresent professionals who are more interested in, experienced with, or favorable toward AI, while the perspectives of nonparticipants and less AI-engaged practitioners remain unobserved. Cross-sector and cross-regional heterogeneity and statistical controls do not eliminate this self-selection problem, and the direction of bias in the coefficients cannot be determined. The findings should therefore be generalized analytically and cautiously, not treated as population-representative estimates.

Fourth, AI-augmented procurement systems vary widely in scope and autonomy, so respondents may have had different reference systems in mind despite the definitional framing. The qualitative phase drew on a limited number of cases, and the survey relied on cross-sectional self-reports. Future research should use standardized vignettes, longitudinal designs, matched dyads, and field experiments on socio-affective signaling, human-AI override practices, and interaction effects among trust-by-design levers.

Ultimately, trust in AI-augmented negotiations is neither automatic nor static—it must be designed, governed, and calibrated. Building trustworthy AI in B2B procurement requires technical competence, ethical visibility, and meaningful human control. Organizations that align these elements may be better positioned to support appropriate reliance on AI assistance; whether this alignment also improves fairness, cooperation, and resilience in buyer–supplier relationships requires direct dyadic evidence.

## Data Availability

The datasets presented in this article are not readily available because the dataset contains confidential organizational information and responses from human participants. To protect anonymity and comply with institutional ethics approval and the EU General Data Protection Regulation (GDPR), raw data cannot be publicly shared. Anonymized subsets may be provided upon reasonable request to the corresponding author, subject to ethical review and data-sharing agreements. Requests to access the datasets should be directed to ramejri@uj.edu.sa.
