# Supplementary material for: Trust by design in AI-augmented procurement systems: the roles of explainability, governance, and human oversight
Source: Front Artif Intell. 2026 Jul 31;9:1752349. doi: 10.3389/frai.2026.1752349 (PMC13473399; doi:10.3389/frai.2026.1752349)
Supplement: Supplementary file 1 [file Supplementary_file_1.docx]

**Supplementary Material**

**Appendices A and B**

Ready-to-publish supplementary material supplied separately from the main manuscript. This file contains no article title, author list, affiliations, correspondence details, line numbering, or tracked changes.

# Appendix A. Measurement instrument and diagnostic material

## A1. Survey instrument (summary)

All items were measured on five-point Likert scales (1 = strongly disagree; 5 = strongly agree), except the control variables described below.

**AI Use (AIU)** - Adapted from negotiation-support literature and information-systems transparency/usage research.

- AIU1 - Our procurement team regularly uses AI-based tools during supplier negotiations.
- AIU2 - AI systems are integrated into most stages of our negotiation process.
- AIU3 - AI outputs influence key negotiation decisions.

**Transparency / Explainability (XAI)** - Adapted from Wanner, Herm, Heinrich, and Janiesch (2022), Coussement, De Caigny, and Benoit (2024), and Riedl (2022).

- XAI1 - The AI system provides clear reasoning for its recommendations.
- XAI2 - I can easily understand how the AI reaches its negotiation outcomes.
- XAI3 - Decision rules used by the AI are transparent to users.
- XAI4 - The system allows users to trace data sources or parameters used.

**Ethical Governance (EG)** - Adapted from Reier Forradellas and Garay Gallastegui (2021).

- EG1 - Our organization has formal oversight mechanisms for AI ethics.
- EG2 - AI negotiation systems are subject to compliance or audit reviews.
- EG3 - Users are informed about data-use policies and accountability procedures.
- EG4 - There are clear protocols for reporting or correcting unethical AI outcomes.

**Relational Design (RD)** - Human-in-the-loop features adapted from case insights, trust/automation literature, and negotiation-support design.

- RD1 - The AI interface enables direct human oversight during negotiations.
- RD2 - Human managers can override or adjust AI decisions when necessary.
- RD3 - The system maintains interpersonal cues (tone, feedback, responsiveness).
- RD4 - AI features are designed to complement, not replace, human interaction.

**Perceived Efficiency (PE)** - Adapted from Rai, Patnayakuni, and Seth (2006) and Wiengarten et al. (2011).

- PE1 - AI negotiation tools speed up decision-making.
- PE2 - AI helps reduce negotiation costs or cycle times.
- PE3 - AI enhances the consistency and quality of negotiated outcomes.

**AI System Trust (AST)** - Adapted from McKnight, Choudhury, and Kacmar (2002) and Gefen, Karahanna, and Straub (2003).

- AST1 - I trust the recommendations generated by the AI negotiation system.
- AST2 - I believe the AI system acts in good faith during negotiations.
- AST3 - I feel confident that the AI system will protect sensitive information.
- AST4 - Overall, I trust this AI system to support fair and effective negotiations.

### Prospective extension (not estimated in the present model)

The following items are documented for transparency and future replication. They were not included in the current estimations or tables.

- RD5 - Human gate reviews validate key AI proposals before supplier disclosure.
- RD6 - Roles and responsibilities for human override are explicitly defined.
- RD3-R - During interaction, the interface preserves human cues (identity/role, confirmations, and a human contact channel).
- RD7 - Transitions between AI and human agents are clearly signposted and archived.
- RD8 - AI justifications are provided in context-appropriate natural language.
- UI1 - The interface is easy to use.
- UI2 - Visual presentation is clear.
- UI3 - System responsiveness is adequate.

### Control variables

- Firm size: number of employees (small = fewer than 500; medium = 500-5,000; large = more than 5,000).
- Digital maturity: self-reported level of AI integration (1 = low; 5 = high).
- Negotiation frequency: average number of supplier negotiations per month.
- Region: categorical (EU = 1; ASEAN = 2).

Instrument notes: Items were translated and pretested with 15 procurement professionals for clarity. The final instrument was reviewed by three academic experts in information systems and supply chain management. All constructs were treated as reflective indicators in the SEM model estimated in AMOS 28.

## A2. Measurement and correlation diagnostics

### Table A1. Cross-loadings and reliability diagnostics (CFA, standardized loadings)

| **Construct / item** | **Loading** | **SE** | **t-value** | **CR** | **AVE** | **alpha** |
| --- | --- | --- | --- | --- | --- | --- |
| **AI Use (AIU)** |  |  |  | 0.88 | 0.64 | 0.85 |
| AIU1 | 0.82 | 0.04 | 20.5 |  |  |  |
| AIU2 | 0.84 | 0.03 | 23.1 |  |  |  |
| AIU3 | 0.79 | 0.05 | 18.9 |  |  |  |
| **Transparency / Explainability (XAI)** |  |  |  | 0.91 | 0.63 | 0.89 |
| XAI1 | 0.81 | 0.04 | 21.4 |  |  |  |
| XAI2 | 0.83 | 0.04 | 22.8 |  |  |  |
| XAI3 | 0.78 | 0.05 | 18.3 |  |  |  |
| XAI4 | 0.80 | 0.04 | 19.7 |  |  |  |
| **Ethical Governance (EG)** |  |  |  | 0.87 | 0.58 | 0.83 |
| EG1 | 0.77 | 0.05 | 17.9 |  |  |  |
| EG2 | 0.80 | 0.04 | 20.6 |  |  |  |
| EG3 | 0.75 | 0.05 | 16.8 |  |  |  |
| EG4 | 0.79 | 0.04 | 18.9 |  |  |  |
| **Relational Design (RD)** |  |  |  | 0.88 | 0.61 | 0.85 |
| RD1 | 0.82 | 0.04 | 20.8 |  |  |  |
| RD2 | 0.85 | 0.03 | 22.7 |  |  |  |
| RD3 | 0.79 | 0.04 | 19.5 |  |  |  |
| RD4 | 0.77 | 0.05 | 18.3 |  |  |  |
| **Perceived Efficiency (PE)** |  |  |  | 0.89 | 0.62 | 0.87 |
| PE1 | 0.83 | 0.04 | 21.2 |  |  |  |
| PE2 | 0.85 | 0.03 | 22.6 |  |  |  |
| PE3 | 0.78 | 0.05 | 19.1 |  |  |  |
| **AI System Trust (AST)** |  |  |  | 0.93 | 0.66 | 0.91 |
| AST1 | 0.84 | 0.03 | 23.8 |  |  |  |
| AST2 | 0.86 | 0.03 | 24.5 |  |  |  |
| AST3 | 0.82 | 0.04 | 21.9 |  |  |  |
| AST4 | 0.80 | 0.04 | 19.8 |  |  |  |

Notes. CR = composite reliability; AVE = average variance extracted; alpha = Cronbach's alpha. All loadings were at least 0.70; all t-values exceeded 15.0 (p < 0.001).

### Table A2. Inter-construct correlations and discriminant validity

| **Construct** | **AIU** | **XAI** | **EG** | **RD** | **PE** | **AST** | **sqrt(AVE)** |
| --- | --- | --- | --- | --- | --- | --- | --- |
| AI Use (AIU) | 0.80 |  |  |  |  |  | 0.80 |
| Transparency / Explainability (XAI) | 0.48 | 0.79 |  |  |  |  | 0.79 |
| Ethical Governance (EG) | 0.44 | 0.46 | 0.76 |  |  |  | 0.76 |
| Relational Design (RD) | 0.41 | 0.49 | 0.42 | 0.78 |  |  | 0.78 |
| Perceived Efficiency (PE) | 0.56 | 0.51 | 0.39 | 0.46 | 0.79 |  | 0.79 |
| AI System Trust (AST) | 0.37 | 0.64 | 0.48 | 0.52 | 0.44 | 0.81 | 0.81 |

Notes. Diagonal elements are the square root of AVE; off-diagonal values are latent correlations. The Fornell-Larcker criterion was satisfied and all HTMT ratios were below 0.85.

## A3. Multicollinearity and common-method diagnostics

| **Variable** | **VIF** | **CMV marker loading** | **Harman single-factor variance (%)** |
| --- | --- | --- | --- |
| AIU | 2.41 | 0.07 | 28.3 |
| XAI | 2.52 | 0.06 | - |
| EG | 2.18 | 0.05 | - |
| RD | 2.35 | 0.06 | - |
| PE | 2.01 | 0.08 | - |
| AST | 2.45 | 0.07 | - |

Notes. All VIF values were below 3.0; latent-method-factor loadings were below 0.10; single-factor variance was below 30%, indicating no dominant common-method factor.

## A4. Qualitative-to-quantitative audit trail (illustrative mapping)

| **Qualitative theme** | **Illustrative evidence** | **Construct / mechanism** | **Survey operationalization** | **Hypotheses informed** |
| --- | --- | --- | --- | --- |
| Need for defensible recommendations | Participants emphasized the need to justify AI outputs to internal stakeholders and in audits. | XAI -> perceived competence and procedural fairness | XAI1-XAI4: reasoning clarity, understandability, rule transparency, traceability | H2; mediation interpretation |
| Depersonalization / relational displacement | Automation reduced interpersonal exchanges and socio-cognitive cues. | HIL/RD -> controllability and relational legitimacy | RD1-RD4: oversight, override, socio-affective cues, complementarity | H1; H4 |
| Governance visibility gaps | Ethics/compliance structures existed but were not visible to users. | Ethical governance -> integrity and accountability | EG1-EG4: oversight, audit reviews, data-use policies, reporting protocols | H3 |
| Power asymmetry and fairness concerns | Unequal AI access raised fairness concerns in tenders and cross-border deals. | Rule transparency and contestability as boundary condition | Reflected across XAI/EG/RD and discussed as a contextual condition | H1-H4 contextualization |

# Appendix B. Multi-group invariance and robustness tests

## Table B1. Measurement invariance across EU and ASEAN groups

| **Model** | **chi-square/df** | **CFI** | **TLI** | **RMSEA** | **delta CFI** | **delta RMSEA** | **Decision** |
| --- | --- | --- | --- | --- | --- | --- | --- |
| Configural (no constraints) | 1.92 | 0.940 | 0.925 | 0.043 | - | - | Baseline accepted |
| Metric (loadings constrained) | 1.95 | 0.938 | 0.926 | 0.043 | -0.002 | 0.000 | Invariant |
| Scalar (loadings + intercepts) | 1.97 | 0.932 | 0.921 | 0.044 | -0.006 | +0.001 | Invariant (delta CFI <= 0.010; delta RMSEA <= 0.015) |

Interpretation. Configural, metric, and scalar invariance were supported, allowing meaningful comparison of structural paths across regions.

## Table B2. Robustness checks

| **Estimation** | **chi-square/df** | **CFI** | **TLI** | **RMSEA** | **SRMR** | **Comment** |
| --- | --- | --- | --- | --- | --- | --- |
| ML (default) | 1.94 | 0.936 | 0.921 | 0.042 | 0.048 | Baseline SEM |
| DWLS (ordinal) | 1.91 | 0.945 | 0.931 | 0.041 | 0.046 | Comparable fit |
| PLS-SEM replication | - | R2_PE = 0.35 | R2_AST = 0.63 | - | - | Same path signs; no suppression |

Notes. DWLS = diagonally weighted least squares, robust to ordinal items. The PLS replication used SmartPLS 4 and confirmed stability of the path signs.

## Table B3. Code and data availability summary

| **Analysis** | **Software / syntax** | **Notes** |
| --- | --- | --- |
| CFA / SEM | AMOS 28 (.amw) | Complete model with mediations and quadratic term |
| Bootstraps (5,000 BCa) | AMOS plug-in / R lavaan | Used for indirect and non-linear tests |
| Invariance | R semTools | Configural-metric-scalar model comparison |
| Robustness | SmartPLS 4 | PLS-SEM cross-validation |
| Dataset | Anonymized .sav / .csv | Available upon reasonable request, subject to ethical review, data-sharing agreements, and GDPR compliance |

**End of Supplementary Material**
